# Cancer Services and Their Initiatives to Improve the Care of Indigenous Australians

**DOI:** 10.3390/ijerph15040717

**Published:** 2018-04-11

**Authors:** Emma V. Taylor, Margaret M. Haigh, Shaouli Shahid, Gail Garvey, Joan Cunningham, Sandra C. Thompson

**Affiliations:** 1 Western Australian Centre for Rural Health, The University of Western Australia, 167 Fitzgerald Street, Geraldton, WA 6530, Australia; margaret.haigh@uwa.edu.au (M.M.H.); S.Shahid@curtin.edu.au (S.S.); sandra.thompson@uwa.edu.au (S.C.T.); 2 Centre for Aboriginal Studies, Curtin University, Kent Street, Perth, WA 6102, Australia; 3 Menzies School of Health Research, Charles Darwin University, Darwin, NT 0811, Australia; Gail.Garvey@menzies.edu.au (G.G.); joan.cunningham@menzies.edu.au (J.C.)

**Keywords:** Aboriginal and Torres Strait Islander, Indigenous Australians, cancer, cancer services, cancer care, treatment, cultural safety

## Abstract

Indigenous Australians continue to experience significantly poorer outcomes from cancer than non-Indigenous Australians. Despite the importance of culturally appropriate cancer services in improving outcomes, there is a lack of awareness of current programs and initiatives that are aimed at meeting the needs of Indigenous patients. Telephone interviews were used to identify and describe the Indigenous-specific programs and initiatives that are implemented in a subset of the services that participated in a larger national online survey of cancer treatment services. Fourteen services located across Australia participated in the interviews. Participants identified a number of factors that were seen as critical to delivering culturally appropriate treatment and support, including having a trained workforce with effective cross-cultural communication skills, providing best practice care, and improving the knowledge, attitudes, and understanding of cancer by Indigenous people. However, over a third of participants were not sure how their service compared with others, indicating that they were not aware of how other services are doing in this field. There are currently many Indigenous-specific programs and initiatives that are aimed at providing culturally appropriate treatment and supporting Indigenous people affected by cancer across Australia. However, details of these initiatives are not widely known and barriers to information sharing exist. Further research in this area is needed to evaluate programs and initiatives and showcase the effective approaches to Indigenous cancer care.

## 1. Introduction

Cancer is one of the leading causes of death for Aboriginal and Torres Strait Islander (hereafter referred to as Indigenous) Australians [1,2]. Despite the advances in cancer detection and treatment techniques, and overall improvements in cancer outcomes in Australia, Indigenous Australians continue to experience significantly poorer outcomes than non-Indigenous Australians for an equivalent stage of disease [3,4,5]. Ethnic and racial health disparities in cancer survival have also been found in countries comparable with Australia, with a similar pattern of disproportionate cancer mortality in Indigenous populations relative to non-Indigenous populations being observed in countries such as New Zealand, Canada, and the United States of America (USA), which share similar histories of colonisation and marginalisation [6,7,8,9]. A number of factors have been identified as contributors to poorer Indigenous cancer outcomes, including lower participation in screening programs, later stage at diagnosis, the presence of other chronic diseases, and lower uptake and completion of cancer treatment [5,8,10,11,12]. 

It is widely recognised that, as with health services in general, there is a lack of culturally appropriate cancer services to engage Indigenous peoples. Fear or lack of trust of mainstream health facilities, lack of understanding or respect being shown by health care providers, poor communication by health care providers, fatalistic or differing cultural beliefs about cancer, and logistical difficulties in accessing screening and treatment services have been identified as inhibiting Indigenous people’s engagement with cancer care [12,13,14,15,16,17,18]. It has been noted that attention should focus on how cancer service providers deliver services to their Indigenous patients and on ways in which they can improve to better meet the needs of Indigenous peoples [12,13,17]. In Australia, the *National Aboriginal and Torres Strait Islander Cancer Framework* has identified that “culturally safe services and a culturally competent workforce… are core requirements for improving cancer outcomes” [19] (p. 15), highlighting the essential role that cancer service providers have in encouraging and maintaining Indigenous Australians’ attendance at cancer services to gain the benefits of modern cancer treatments. 

In the USA, there have been a number of evaluations of cancer service initiatives designed for American Indians (AI), with articles evaluating two patient navigator programs [20] and six cancer survivorship programs [21]. However, in Australia, there has been limited formal evaluation of cancer service delivery initiatives for Indigenous Australians. One of the few studies carried out examined cancer support services in Queensland [22]. Other studies measured the satisfaction levels of Indigenous patients and their family members with various aspects of a tele-oncology service in North Queensland [23] and compliance with radiotherapy treatment in the Northern Territory [24]. In addition, there appears to be a lack of awareness amongst cancer service providers about what other services are doing and which services are delivering culturally appropriate care [25]. Furthermore, many cancer service providers have indicated that they lack the “know how”, or their services do not have the specific policies and/or Indigenous-specific programs that are required to bring about change [16,22].

Given the dearth of published information and the lack of awareness of what cancer services are doing to meet the needs of Indigenous patients, there is an evident need to learn from cancer services that have programs and initiatives for their Indigenous cancer patients and their families. This need is perhaps the greatest for cancer care professionals that are located in regional and remote locations who often have a larger proportion of Indigenous cancer patients [1], but are geographically isolated from other services and often lack the professional development opportunities of their metropolitan-based colleagues [26]. 

This study was conducted as part of a broader investigation undertaken to identify and describe cancer services providing treatment to Indigenous cancer patients in Australia. The aim of the present study was to learn, using telephone interviews, what Indigenous-specific programs and initiatives have been implemented in a subset of the services who participated in an earlier national online survey of cancer treatment services [25]. In addition, we explored the innovation enablers for these service providers and identified their suggestions for future improvements in order to better meet the needs of their Indigenous cancer patients. Although none of the programs or initiatives has been evaluated as part of this or any other study, this information has the potential to assist cancer service providers to identify gaps in current services and to plan new service delivery initiatives.

## 2. Materials and Methods 

Ethics approvals were obtained from the Human Research Ethics Committees of University of Western Australia, the Western Australian Aboriginal Health Ethics Committee (WAAHEC) (ethical approval number was 483) and the Western Australian Country Health Service (WACHS) (ethical approval number was 2013:20), as well as multiple other ethics committees. 

### 2.1. Recruitment of Participants

From the 58 public hospitals that participated in the earlier online survey [25], 18 services of interest were identified for inclusion, based upon their reporting specific programs, policies, or resources in place that were aimed at meeting the needs of Indigenous cancer patients and their families. The 18 identified services were contacted by telephone and were invited to participate in a follow-up telephone interview. Fourteen of the 18 identified services participated in interviews. Two services declined to participate and two services were not available for interview during the data collection period. 

### 2.2. Data Collection

Between March and October 2015, 15 telephone interviews and two face-to-face interviews were conducted with a range of management, support, and clinical staff from the participating services. The interviews ranged from 20 to 90 min, and averaged 50 min duration. In three instances, two participants were interviewed together because the participants felt that a joint interview would provide more complete information. At two sites, multiple interviews were conducted when it was identified that information could be provided by participants with different roles or based at different locations of the service. 

The interviews were guided by seven broad open-ended questions (Table 1). All of the interviews were recorded, apart from one in which the participant declined to be recorded but consented to interview with extensive notes being taken. Written or oral (recorded) consent was provided by all of the participants prior to the interview.

### 2.3. Data Analysis

We utilised a tape and notes based approach [27]. The interviewer (EVT) listened to the interview recordings and made in-depth summaries, capturing the central points that were discussed. In addition, key issues of the discussion were transcribed verbatim. Two other co-authors (Margaret M. Haigh and Sandra C. Thompson) and a member of the wider research team independently reviewed a selection of the interviews and any divergence with the summaries was discussed and reviewed until a consensus was reached. Each interview summary was sent to the participant(s) for comments and validation. A number of participants made changes to their interview summary and these amended versions became the final copies. 

All of the summaries were manually coded and independently analysed using both an inductive and deductive approach. Initial exploratory analysis of the data occurred by examining the data without using a guiding framework to identify some key themes in relation to activities, initiatives, or policies that were implemented. Then, the data were examined using Levesque’s five dimensions of accessibility [28] from which further themes emerged. This output was subsequently analysed using the *National Aboriginal and Torres Strait Islander Cancer Framework* [19], which was published after the study had commenced. This Framework guided the latter part of the process, and, from this subsequent analysis, additional themes in relation to activities, initiatives, or policies that were implemented emerged. The themes reported in the first part of the Results section below are categorised broadly in line with the priorities that were identified in the Framework.

The interview summaries were then analysed to explore the catalysts for changes to service delivery, suggestions for ways in which services could improve to better meet the needs of Indigenous patients, and how respondents would rate their own performances in comparison to other services. This stage of the analysis was done without using a guiding framework; instead, an inductive, exploratory approach was adopted to identify the emerging themes.

## 3. Results

### 3.1. Characteristics of Participating Cancer Service Providers

Representatives of fourteen services participated in interviews. Eight of the 14 services (57%) were located in regional or remote areas (Table 2). All states and territories, apart from the Australian Capital Territory (ACT) and Tasmania, were represented. Most of the services were cancer centres, either standalone or co-located within a hospital. Others represented cancer centres across multiple hospitals within a health district, a cancer nurse coordination service, and a service improvement unit. Some services from which the participants were interviewed had a limited number of Indigenous-specific programs and policies, whereas others had many initiatives in place. All but one reported a case load in excess of five Indigenous patients per annum, with some treating more than 50 Indigenous patients.

A total of 20 staff members (16 female and four male) were interviewed. Participants were in diverse roles, including a range of managerial positions, cancer care coordinators, nurses, project officers, and one Aboriginal Liaison Officer. Five participants identified themselves as Indigenous Australian.

### 3.2. Key Activities, Initiatives or Policies Implemented to Ensure Culturally Appropriate Treatment and Support

The findings that are reported below are categorised broadly in line with the priorities that were identified in the *National Aboriginal and Torres Strait Islander Cancer Framework*.

#### 3.2.1. Trained Workforce with Effective Communication Skills

Providing a suitable workforce, appropriately trained, and having effective cross-cultural communication skills, was identified by several participants as an important part of their efforts to provide culturally appropriate treatment and support to Indigenous people that are affected by cancer. Ten of the participating services reported employing one or more Indigenous staff members. Indigenous staff roles included Indigenous Liaison Officers (ILOs), Cancer Care Coordinators, project officers and frontline administrative staff. The perceived benefits of having a cancer-specific Indigenous staff role were seen in enhanced engagement and follow up of Indigenous patients, as articulated by one participant: “*Having [the Aboriginal Health Services Officer] there to do the liaison between community, the family and us, to actually physically get them in here and through the doors means that there’s probably some patients that we’re seeing that we wouldn’t have seen before. They would have just fallen off the radar somewhere.*” (Head—Social Work Department, ID 16).

A smaller number of services (*n* = 4) described using a multidisciplinary team approach to plan and deliver cancer care to their Indigenous patients. Several stressed the importance of having an ILO being included as an active member of the team, as highlighted by one Indigenous participant: “*We’re very much a part of team. I don’t know how they’d go without us really…. They always look to us around what’s going on….*” (Indigenous Cancer Co-ordinator, ID 1).

Six of the services reported having educational programs as part of professional development specifically for their Indigenous Health Workers (IHWs). These programs included cancer education sessions (for example, on how to care for cancer patients in the community); site visits to cancer treatment centres (including some based in large metropolitan centres) to understand what patients experience and to network with staff; and, placements with other cancer services. 

In addition to employing Indigenous staff, efforts to provide culturally appropriate treatment and support for Indigenous patients and families were reinforced with cultural awareness training; this was mandatory at 12 of the 14 (86%) respondent’s services, although it was unclear whether this applied to all staff. The cultural awareness training programs that were described varied in delivery and duration, from a 2 h webinar to a one-day face-to-face workshop. One health district even incorporated responsibility for implementing lessons from the training into employees’ performance reviews once the employee had attended training. 

#### 3.2.2. Best Practice Care

Providing the best practice care was acknowledged by many participants as another consideration in their endeavours to provide culturally appropriate treatment and support. Nine services described increased flexibility in their clinical practice in attempts to meet the cultural and family needs of their Indigenous cancer patients. Initiatives that were identified included offering outdoor consultations, providing a shorter treatment program, providing access to clinical staff of the appropriate gender and grouping Indigenous patient appointments together. One participant explained: “*… [the Indigenous cancer patients] may not come into the hospital environment at all… we will sit outside and just yarn… [it was] a change of culture for all of us… leaving our documentation behind. Not having our papers, just going and really hearing what patients are [telling us].*” (Rural Cancer Nurse Coordinator, ID 18).

Nine services described changes to the physical environment, either planned or already made, which were aimed at improving the cultural safety of the treatment centre when striving to provide best practice care. Examples included flying the Aboriginal flag, installing artwork created by local Indigenous artists, and displaying a welcome statement in the entrance in local language. Some centres had consulted with the local Indigenous community when planning to build a new cancer centre or renovating an existing centre, and then incorporated suggested features into the structure of the building. Examples provided included outdoor waiting areas and efforts to “bring the outside in” by installing windows with views of nature, a colour scheme that represented the local landscape, and fish tanks to represent the local waterways. One centre had installed a light box above the LINAC (radiotherapy machine): “*When you lay under it, it’s actually like you’re lying under a tree, looking through the branches to the sky.*” (Project Officer Aboriginal Cancer Services, ID 7).

Another initiative that was aimed at delivering best practice care was having processes or positions in place to support Indigenous patients throughout their cancer journey (not just while undergoing treatment). This was reported by seven services; all treated larger numbers (>50) of Indigenous patients per annum or had a minimum of 10% Indigenous patients. Two services employed Indigenous Cancer Care Coordinators whose role was to support the patients during their entire time at the cancer service. One metropolitan service had developed an Indigenous Cancer Patient Pathway that incorporated cultural needs to be considered at each stage, for example, the level of family involvement during the treatment stage. Involving the patient’s family was mentioned by seven services, both remote and metropolitan. Usually family was involved and informed by holding one or more family meetings, either in person or via videoconference, prior to commencing treatment and sometimes during treatment. Very few services mentioned the survivorship element of the patient journey; however, one remote service was developing a formalised Survivorship Program at the time of interview, which would include a one-on-one counselling session about the patient’s needs after completing treatment.

Ten services stressed the importance of working with local health networks when striving to deliver best practice care. Organisations mentioned included Aboriginal Community Controlled Health Services (ACCHS), Primary Health Network, Cancer Council, palliative care organisations, and, more occasionally, Indigenous community representatives. The purpose of these partnerships was to break down silos, pool resources, and coordinate care to Indigenous patients to ensure that: “*… the right service is contacting the patient at the right time*” (Rural Cancer Nurse Coordinator, ID 18).

One participant in a regional service described how the local health services had formed an Aboriginal Cancer Network with the aim of improving cancer outcomes for Indigenous people in relation to prevention, screening, treatment, survivorship, and palliative care. The Network worked on projects under these headings, focusing on how to improve access, remove barriers, and improve survival. It was not clear whether any of the initiatives had been evaluated. Activities undertaken by the Network included a Well Women’s Workshop for breast cancer. The Network relied on passionate, committed members who worked on projects in addition to their core work, and it was acknowledged that this was likely to be challenging to sustain long-term.

Half of the services that were interviewed (most of whom treated in excess of 50 Indigenous patients per annum or whose case load was a minimum 10% Indigenous) offered outreach services in efforts to improve care. Three services had a formal or frequent outreach service and another four had more limited or ad-hoc outreach. Examples of outreach included a medical oncologist that was flying to a remote site on a regular basis to conduct clinics, and home visits by nurses or social workers. Informants in centres that were not providing outreach commented on capacity as an issue. However, a participant from a remote service without the capacity to undertake site visits, emphasised the importance of clinical staff visiting remote Indigenous communities in order to gain a better understanding of patients’ living conditions, which might then result in more realistic post-treatment care instructions.

Four services had a formalised telemedicine or tele-oncology service; all of them were located in outer regional or remote areas and used telemedicine to support initiatives in remote areas, such as running oncology clinics, supporting IHWs, delivering education and training to health professionals, and holding family meetings with patients and their families. The perceived advantages of telemedicine that were described by participants included less patient travel for treatment or follow-up consultations (with consequent improvements in compliance); reduced wait times for patients; increased education and orientation pre-attendance; and, more efficient use of oncologists’ time. In addition to using the video link for the purpose of telemedicine, in one service, patients were also able to use it to keep in touch with their families whilst undergoing treatment.

#### 3.2.3. Knowledge, Attitudes and Understanding of Cancer

Improving the knowledge, attitudes, and understanding of cancer by individuals, families, carers, and community members was identified by many participants as an important factor in their endeavours to provide culturally appropriate treatment and support. The most frequently reported initiatives in this regard involved engaging communities, developing in-house resources, and using patients as advocates. Examples of community engagement (*n* = 10) included a cancer service running (or contributing to) community education sessions and cancer centre open days to raise awareness of cancer or the centre. Ten services reported having created in-house educational resources that were targeted specifically at their local Indigenous community, including brochures and booklets, posters, videos, magnets, a cancer services directory, and even pins. Resources were created in-house, ideally with input from the local Indigenous community and using local language where appropriate, were reported as being more appropriate and useful than educational materials that were created by external organisations. However, this was contingent on staff and financial resources being available. A small number of the regional cancer services (*n* = 4) had engaged past patients to be advocates for the service, often with survivors creating videos of their experiences.

#### 3.2.4. Prevention and Screening Programs

Two services had recently started focusing on prevention, with one starting an anti-smoking program with a remote Indigenous community. The importance of cancer centres becoming involved with prevention was articulated by one senior participant, who stated that running a prevention program is: “*…hard but we have to do it... at the moment prevention is left to primary care… cancer centres need to take on tackle prevention. We all see patients and we can do primary and secondary prevention… and really should not need extra funding*”*. *(Director Medical Oncology, ID 17).

Four services, mostly being located in regional areas, ran screening programs for the local Indigenous communities. All of the screening programs that were described were for women’s cancers, such as breast screening (most commonly reported) and pap smears, although one service did plan to run a workshop on prostate cancer. 

#### 3.2.5. Capacity to Deliver Quality Services 

Strengthening the capacity to deliver quality, integrated services that meet the needs of Indigenous people was identified as key when aiming to provide culturally appropriate treatment and support. Four services described activities to support continuous improvement. Continuous improvement activities included surveying past patients and obtaining feedback from open days and community education sessions. One service utilised the *Aboriginal and Torres Strait Islander Patient Quality Improvement Framework and Toolkit for Hospital Staff* (AQIFTHS), which is a resource “*designed to provide a systematic approach to improving Aboriginal health service delivery in hospitals*” [30] (p. 17).

Initiatives or policies to ensure that Indigenous patients were correctly identified were reported by six of the services in efforts to strengthen their capacity to deliver better outcomes. This usually occurred by encouraging, or mandating, frontline staff to ask all patients whether they identified as Aboriginal or Torres Strait Islander at admission, or, failing this, subsequent presentation. Several services reported providing specific “identification training” to frontline staff on how to ask the question respectfully. All of the services reported that their efforts in this area improved data quality, with success being measured by a high concordance in identification when audited. Some respondents reported that the processes had become “embedded” so that regular reminders to frontline staff were no longer necessary.

### 3.3. Catalysts for Change

Most of the participants identified multiple catalysts for having changed, in their opinion, the way that their service worked with Indigenous patients, health services, and communities over the last five years. Half of the services reported receipt of funding, either for a specific project (such as the creation of cancer resources) or a new role (for example, Aboriginal Cancer Care Coordinator), as catalysts for change: “*…without that injection of funds … we would still be going round the hamster wheel, not really sure how to start. It [funding] was critical in creating a space to allow something to happen.*” (Head—Social Work Department, ID 16)*.*

Other perceived catalysts for change included the involvement in an event or project, such as an open day, conference, or being involved in research, such as a study to evaluate the Supportive Care Needs Assessment Tool for Indigenous People (SCNAT-IP) [31]. Increased or mandatory cultural awareness training was also felt to stimulate change through a shift in organisational culture. Policy changes at the state or federal level (such as Closing the Gap [32]) were also thought to be important change drivers for individual health districts or organisations, as were the establishment of networks and relationships with local services (such as the local ACCHS). Other perceived catalysts for change were the presence of strong advocates, improvements in Indigenous patient identification rates, results from patient surveys, and the building of a local dedicated cancer care unit. 

### 3.4. Suggestions for Future Improvements

Although participants highlighted initiatives that they had undertaken with a view to better accommodating the needs of Indigenous patients, they made a large number of suggestions about how their service could be improved further. These included listening to their Indigenous patients, and adapting their service accordingly. Many suggestions were related to improving the cultural competency of their workforce through increasing or improving cultural awareness training for staff. One participant noted that additional and ongoing training was required due to an underlying lack of understanding of the history of Indigenous Australians and the inequities they experience: “*I still hear at our high level meetings, the people that are supposed to be pushing this, they say things like ‘I didn’t cause this problem’, ‘I don’t understand why I’ve got to say sorry’, ‘I treat everybody the same*’.” (District Cancer Clinical Nurse Consultant, ID 4).

Other suggestions were to hire more Indigenous staff to do activities outside the usual scope of clinical staff (such as prevention, engagement, follow-up, and site-visits); and, to facilitate patient accessibility to treatment by providing telemedicine or outreach services and by improving accommodation and transport.

### 3.5. Comparison to Other Services and Awareness of Best Services

When asked to expand on how their service compared with other cancer services in meeting the needs of Indigenous people with cancer, almost half of the respondents thought that their service was doing better than others (*n* = 6). Five services were not sure how their service compared, indicating they were not sufficiently aware of what other services are doing in this field. 

The respondents listed a total of 21 “best services”, in terms of treating Indigenous patients with cancer (the question did not specify whether their own service could be included but two services did self-nominate). The entities that were identified consisted of a diverse range of services across Australia including hospitals, cancer centres, Aboriginal Community-Controlled Health Services (ACCHSs), Cancer Councils, and breast screening services. Among the reasons given why a service was identified as “doing best” were staff (particularly access to Indigenous staff), resources, tele-oncology, and specific systems or processes targeted to meet the needs of Indigenous people. Nine of the 14 participating services were included amongst the 21 nominated “best services”.

## 4. Discussion

This study provides a description of key activities, initiatives, and policies that were implemented by the services who participated in our study, all of which had previously indicated that they were actively making an effort to meet the needs of their Indigenous patients. Our study also explored the catalysts for changes to service delivery, suggestions for ways in which services could improve to better meet the needs of Indigenous patients, and how respondents would rate their own performance in comparison to other services. The services were located across Australia, in most states and territories and in a variety of locations with respect to size and rurality. Our study confirmed that all services were actively making efforts to meet the needs of their Indigenous patients, but, as expected, the participants were at different stages on their journey to providing culturally appropriate health care. 

Our study found that having a trained workforce with effective communication skills is seen as critical when striving to deliver culturally appropriate treatment and support to Indigenous people that are affected by cancer. This is reinforced by the *National Aboriginal and Torres Strait Islander Cancer Framework*, which identified “a culturally competent workforce” as being essential for improving cancer outcomes [19]. The importance of providing health professionals with appropriate cultural training is noted elsewhere, both in Australia and internationally [20,33,34,35,36,37]. This is underlined by our study, which confirmed that a culturally blind “universal” approach to health care persists among some health professionals who believe that by “treating everyone the same” they are promoting equality [34]. This perspective needs to be systematically addressed both in tertiary health curriculums and in the workplace, to ensure health staff at all levels understand the importance of contextualising Indigenous health within the history of colonisation in Australia and its aftermath [38]. This can be achieved by restructuring tertiary health curriculums to embed the Aboriginal and Torres Strait Islander health Curriculum Framework, providing clinical placements in Aboriginal community-controlled health services for non-Indigenous students, and through mandatory cultural awareness training in the workplace, including cultural mentoring for non-Indigenous health professionals where possible [39]. At a minimum, workplaces should provide on-going cultural safety training to health professionals at all levels, the completion of which should be tracked in performance management requirements [40]. It is essential that training programs ensure health staff understand the importance of acknowledging and working with local cultural protocols, that they cover the wide range of beliefs that Indigenous people have around cancer [41], emphasise differences in communication styles, focus on the need for person-centred care, and encourage the involvement of the patient’s family where appropriate [42]. Involving local Elders and/or Indigenous health professionals in developing and/or delivering training programs is recommended [40]. 

Participating services reported that Indigenous staff members can be especially effective in engaging and following up Indigenous patients, as has been reported previously [43]. The value that is added by Indigenous employees is most evident in the role that they play in helping to negotiate the disparate social and knowledge systems involved when traditional and western health systems interface [44]. Numerous studies, both in Australia and internationally, have highlighted the many practical benefits of having Indigenous health professionals who can provide culturally safe health care to Indigenous patients, advocate for Indigenous patients, and educate other health professionals on the delivery of culturally safe care [44,45,46,47,48,49]. While the importance of having a greater Indigenous presence in the healthcare workforce is undisputed (with a key catalyst being funding for the creation of a new position specifically to work with Indigenous patients), it is not sufficient merely to create such a position. Widespread organisational support is required to ensure that staff undertaking this type of role are provided with adequate training and resources to work effectively as part of an integrated team [38]. This point was frequently underlined by informants who outlined the perceived benefits that are derived from having a well-trained Indigenous health professional involved as an active team member. Allocating sufficient resources—both financial and human—to provide culturally appropriate treatment and support to Indigenous people affected by cancer was emphasised as essential when striving to improve Indigenous cancer outcomes. 

The delivery of culturally appropriate treatment and support is not limited to the care that is provided by Indigenous staff. Non-Indigenous health care providers must understand their shared responsibility in ensuring that Indigenous patients and families feel culturally safe and welcome [38,50], and, from the 15 non-Indigenous participants in our study, there was evidence that these health care providers do so. This is also supported by the positive evaluations of the Walking Forward patient navigation program in South Dakota USA [20], where hospital-based navigators (nurses) worked with community-based navigators to provide the service. Interestingly, despite the strong focus on navigator models in the international literature, there was minimal mention of the navigator model by the participants in our study, reflecting that, unlike in the United States, there has not been widespread adoption of navigators in the Australian context. Service delivery focusing on patient centred care often involves overcoming existing hospital “rules” to ensure that an Indigenous patient feels culturally safe [38]. This practice was confirmed by over half of the services that were interviewed who described needing flexibility in their clinical practice in order to try and meet the needs of their Indigenous cancer patients. Interventions at an interpersonal level must recognise and respect the differences that exist between people from (and within) different cultures [18]. Fear and fatalistic attitudes towards cancer are not uncommon amongst Indigenous people and, when combined with doubt about the efficacy of treatment and fear of hospitals not attuned to their cultural needs, may result in reluctance to access care [18,51]. These factors must be considered when aiming to deliver culturally appropriate treatment and support [52].

While culturally sensitive interpersonal contact is identified as the primary mechanism for facilitating entry into and supporting effective treatment, other interventions at the systemic level must also be considered [38]. A welcoming environment can be reinforced by the design of the building, which can symbolize an acknowledgment of, and respect for, the needs of Indigenous patients [13], and can result in positive health outcomes [38]. A number of participants described changes that were specifically made to the physical environment aimed at improving the cultural safety of the treatment centre and ensuring the provision of best practice care. It was also highlighted that, in some instances, these changes had been made in consultation with the local Indigenous community. Other endeavours undertaken at the systems level in an effort to provide best practice care involved innovations in delivery of clinical practice, such as telehealth and outreach. It has been found that where there is a disadvantaged population with inadequate access to medical care, outreach from a regional centre can provide a more equitable means of service delivery than hospital based services alone [53]. Tele-oncology was also found to be an acceptable model of care for Indigenous patients in one study with high levels of satisfaction being reported by patients, families, and health workers [23].

A number of external factors (for example, the receipt of funding) were identified as having been the catalysts for change in the past. However, it is likely that the key elements that may have been in place for many years, such as the dedication and willingness of staff, were instrumental in bringing about change and that the external opportunities acted as catalysts for the already-primed instigators of change. The suggestions that were made by participants about how their service could improve to better meet the needs of Indigenous patients included providing initiatives to address practical difficulties, such as the provision of transport and accommodation; having a welcoming, supportive hospital environment; employing Indigenous staff; and, developing telehealth and outreach services. Some of these initiatives are already in place in other services and are consistent with recommendations outlined in other studies [13,23,44,53,54]. However, the large number of suggestions made indicates that services are aware that there is scope for improvement in the care that they provide and are keen to deliver change. 

Despite the acknowledged need and the undisputed appetite for improvement, constraints on the capacity of services to deliver change were identified. One constraint is the lack of awareness about what other cancer services have been doing to meet the needs of Indigenous cancer patients. Over one-third of the service representatives (where the service was identified as likely to be a practice leader in the field) were unsure how their service compared to others, and most of the respondents provided vague responses when asked about which were the best performing services. This suggests that cancer service providers may not communicate with each other and have difficulty identifying (and therefore learning from) cancer services that have developed successful Indigenous-specific programs and initiatives. These barriers to information sharing are found at a systemic level and include a lack of formal mechanisms or networks to facilitate learning between services, which is often compounded by geographic isolation that is associated with being located in regional and remote areas. This also presents individual-level barriers to information sharing due to the acknowledged difficulty of accessing professional development when distance is a factor [31]. Furthermore, staff in cancer treatment services have many competing demands on their time, and do not necessarily have the capacity, management support, or even see the need, to spend time on this form of professional development. These barriers to information sharing and professional development are not restricted to staff working in cancer services, nor to those in remote locations, but are found throughout the health professions [25,55].

Barriers to information sharing suggest a need for increased collaboration, networking, and effective partnerships, not just locally, but across cancer services in different locations. Successful partnerships, particularly between Indigenous and mainstream health services, have been identified as crucial because the failure to invest in this relational process can ultimately have negative implications for Indigenous health outcomes [56]. Whilst establishing and developing these relationships have been identified as key in supporting Indigenous cancer patients [22], much work still needs to be done, as is clear from the results of an environmental scan of Cancer Councils, which highlighted challenges in developing and maintaining partnerships with Indigenous organisations [57]. However, it is encouraging that informants from many of the services in our study noted the importance of working together to improve cancer outcomes for Indigenous people and identified the establishment of networks and relationships with local services as having been important change drivers. The *National Aboriginal and Torres Strait Islander Cancer Framework* provides strategic direction to many individuals, communities, and organisations dedicated to improving cancer outcomes amongst Indigenous Australians [19]. By providing high-level guidance and direction, it is hoped that the Framework will facilitate learning and collaboration in the area of Indigenous cancer at a local, regional, jurisdictional, and national level. Moreover, by drawing attention to the subject of Indigenous cancer, the Framework will generate much-needed discussion on this key element of Indigenous health.

### Limitations

Not all of the cancer services participated in the preceding national survey of Australian cancer services from which services of interest were selected to be interviewed. Additionally, four services identified as being of interest did not participate in the interviews, so not all of the services with Indigenous-specific policies, programs, and initiatives have been included in this study. Furthermore, while the interviewer requested to speak to the staff member with the most complete knowledge of initiatives for Indigenous cancer patients, we cannot be sure that this occurred in all instances. It should also be noted that our informants were generally not Indigenous by background. In addition, as information was self-reported by each service, and were not confirmed by independent sources, it cannot be guaranteed that the information was unbiased. 

The initiatives reported in this study were based on reports by the service and have not generally been independently evaluated. While we have every reason to believe that they improve the care experience of Indigenous cancer patients and their families, which in turn could improve outcomes, this was not assessed in our study. 

## 5. Conclusions

The degree to which services are perceived as culturally safe and welcoming towards Indigenous Australians can influence decisions around accessing care. In developing appropriate models of cancer care, attention must focus on how cancer service providers currently deliver services to their Indigenous patients and on ways that they can improve to better meet the needs of Indigenous Australians. Cancer services that are more culturally appropriate and person-centred, and which include and involve Indigenous people, are needed in order to facilitate greater engagement by Indigenous people with cancer. 

Our study has found that there are currently many Indigenous-specific programs and initiatives in place in cancer services throughout Australia that are aimed at providing culturally appropriate treatment and supporting Indigenous people that are affected by cancer. However, the details of these initiatives are not widely known and we have identified some of the reasons behind this lack of awareness. The difficulties that are associated with information sharing demonstrate the need for increased collaboration and effective partnerships, particularly between Indigenous and mainstream health service providers. Encouraging and facilitating co-operation will allow for cancer services to build on their existing initiatives and will enable others to learn from these cancer services. Our study has also highlighted the requirement for further research in this area to evaluate programs and initiatives and showcase in greater detail the more effective approaches to Indigenous cancer care. 

## Figures and Tables

**Table 1:** Guiding interview questions.

1. What do you think your service is doing well to meet the needs of Indigenous patients receiving cancer treatment?
2. If you are offering any outreach services/site visits to Indigenous patients, could you elaborate on those?
3. Compared with other cancer services you are aware of, how would you rate your own performance in meeting the needs of Indigenous people with cancer? Would it be better, about the same or worse? Why do you think that?
4. What service or services in your state or in Australia do you think are doing best in treating Indigenous patients with cancer? Why do you think that?
5. Can you tell us about access to and use of Indigenous health staff within your cancer service to support Indigenous people with cancer while they undertake treatment?
6. Please describe any changes (external and internal to your service) that have occurred over the last 5 years that have changed the way that your service works with Indigenous patients, Aboriginal health services and Indigenous communities?
7. How do you think your service could improve to better meet the needs of Aboriginal patients? What do you think would be needed for this to happen?

**Table 2:** Characteristics of participating cancer service providers.

Characteristics	Number of Services (*n *= 14)
Location by remoteness *	
Major City	6
Inner Regional	4
Outer Regional	3
Remote	1
**Location by State/Territory**	
New South Wales	4
Victoria	3
Western Australia	3
Northern Territory	2
Queensland	1
South Australia	1
**Type of service**	
Cancer centre within a hospital	8
Cancer centres at multiple hospitals within a health district	3
Standalone cancer centre	1
Cancer nurse coordination service	1
Service improvement	1
**No. of Indigenous patients per year**	
50+	6
11–50	5
6–10	2
1–5	1

* Remoteness determined using the Australian Standard Geographical Classification Remoteness Areas, 2006 (ASGC) [29].
